# Upregulation of Protein Phosphatase 2A and NR3A-Pleiotropic Effect of Simvastatin on Ischemic Stroke Rats

**DOI:** 10.1371/journal.pone.0051552

**Published:** 2012-12-10

**Authors:** Minxia Zhu, Jin Wang, Min Liu, Dongshu Du, Chunmei Xia, Linlin Shen, Danian Zhu

**Affiliations:** 1 Department of Physiology and Pathophysiology, Shanghai Medical College, Fudan University, Shanghai, China; 2 Medical College of Tibet University for Nationalities, Xianyang, Shaanxi, China; Virginia Commonwealth University, United States of America

## Abstract

Ca^2+^ influxes are regulated by the functional state of N-methyl-D-aspartate receptors (NMDARs). Dephosphorylation of NMDARs subunits decreases Ca^2+^ influxes. NR3, a novel subunit of NMDARs, also decreases Ca^2+^ influxes by forming new NMDARs with NR1 and NR2. It is meaningful to uncover whether protein phosphatase 2A (PP2A) and NR3A play a role in the protective effect of Simvastatin on ischemic stroke. In the present study, the Sprague-Dawley rats were pretreated with Simvastatin for 7 days before middle cerebral artery occlusion was performed to mimic ischemic stroke. The results showed that Simvastatin decreased brain ischemic infarct area significantly while increasing the expression levels of PP2A and NR3A, thus dephosphorylating the serine sites of NR1 (ser896 and ser897) along with increased enzymatic activities of PP2A. The protein levels of NR3A decreased as the enzymatic activities of PP2A were inhibited by okadaic acid. The results indicated that Simvastatin could protect the cerebrum from ischemic injury through a signaling mechanism involving elevated levels of PP2A and NR3A, and that PP2A might involve in the regulatory mechanism of NR3A expression.

## Introduction

Ischemic stroke, a leading cause of death and disability worldwide, is a major contributor to rising healthcare costs. It has been estimated that in the USA, an individual is afflicted with stroke every 53 seconds with a fatality occurring approximately every 2 minutes, and treatment costs exceeding $65 billion in 2010 [1], [2].

In stroke, excessive extracellular glutamate overstimulates glutamate receptors, initiating an excessive calcium entry mainly through NMDARs, which is the main contributing factor to neuronal excitotoxicity injury during the process of ischemic stroke [3], [4]. Such a role in excitotoxicity has driven the pursuit for antagonists as neuroprotective agents. For their adverse effect on the central nervous system such as hallucinations, a centrally mediated increase in blood pressure and anesthesia, the doses of NMDA antagonists are clinically limited [5] . Statins, 3-hydroxy-3-methylglutaryl coenzyme A reductase inhibitor, whose cholesterol-independent or pleiotropic effect in brain ischemic damage have drawn much focus in clinical trials, for its ability to decrease glutamate, 12/15-lipoxygenase (LOX), p38 mitogen-activated protein kinase (p38MAPK), tumor necrosis factor α (TNF-α), and the ability to increase endothelial nitric oxide synthase (eNOS) and ameliorate potential blood-brain-barrier (BBB) permeability, etc. [6], [7], [8], [9]. When different types of Statins were compared in terms of such parameters of lipophilicity, BBB penetration and cholesterol lowering effect, Simvastatin was more likely to enter endothelial cells by passive diffusion than hydrophilic Statins such as Pravastatin and Rosuvastatin, and it produced the best characteristics in preventing neurodegenerative conditions [10], [11]. Therefore, Simvastatin was employed in the present study to further elucidate its molecular mechanism in ischemic stroke.

Traditional NMDARs are composed of NR1 subunit and different NR2 subunits. NR3A was identified in 1995 as a novel member of the ionotropic glutamate receptor family. In the system of heterologous expression, NR3A entering NR1/NR2 receptors has been reported to significantly reduce Ca^2+^ permeability and Mg^2+^ sensitivity, as the same case in non-NMDA channels [12], [13], [14], [15]. These subtle regulations on NMDARs may be ascribed to a change in channel properties leading to more rapid desensitization kinetics, lower open probability, or lower conductance of the channel [13]. But the mechanism that governs its functional expression remains unclear.

**Figure 1:**
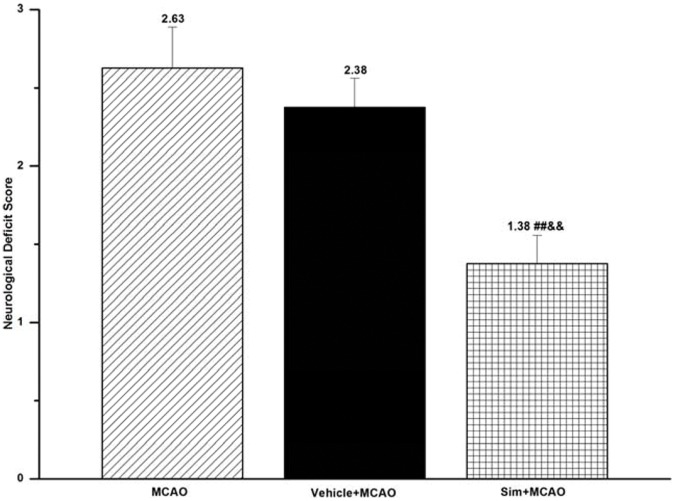
Simvastatin improved neurological deficit of MCAO rats. Statistical analysis of behavior scores, a significantly decreased neurological deficit as a functional outcome observed in sim+MCAO group when compared with that in MCAO and vehicle+MCAO group; values expressed as means ± SEM of 8 rats; ##*P*<0.01, sim+MCAO *vs*. MCAO group; &&*P*<0.01, sim+MCAO *vs*. vehicle+MCAO group. Sim, Simvastatin.

**Table 1:** Neurological Deficit Score of 8 Rats in Each Group.

	1	2	3	4	5	6	7	8	Mean±SEM
Sham	0	0	0	0	0	0	0	0	0±0
MCAO	2	2	2	3	4	2	3	3	2.63±0.26
Vehicle+MCAO	3	2	2	3	2	2	3	2	2.38±0.18
Sim+MCAO	1	1	2	2	1	1	2	1	1.38±0.18^##&&^

## *P*<0.01, sim+MCAO *vs*. MCAO group;

&& *P*<0.01, sim+MCAO *vs*. vehicle+MCAO group. Sim, Simvastatin.

PP2A, a family of serine-threonine phosphatases highly conserved and ubiquitously expressed, is a heterotrimer consisting of a catalytic subunit (C subunit), a structural subunit (A subunit), and a regulatory subunit (B subunit), and is responsible for controlling diverse cellular processes through the negative regulation of signaling pathways initiated by protein kinases [16], [17]. It was reported that the major protein phosphatases, PP1, PP2A and PP2B, suppressed the activities of NMDARs, and in hippocampal neurons, exogenous PP1 or PP2A were employed to depress the open probability of NMDARs single channels; conversely, selective inhibitors of PP1 and PP2A, calyculin A and okadaic acid (OA), exerted an opposite effect with increased NMDARs currents [18]. Skeberdis VA et al. indicated that NMDARs-mediated Ca^2+^ rises were under the control of the protein kinase A (PKA), and PKA blockers markedly inhibited NMDARs-mediated Ca^2+^ rises in activated dendritic spines [19].

The modulation on NMDARs-mediated Ca^2+^ influxes of PP2A and NR3A prompted us to advocate a hypothesis that both two regulatory factors could contribute to the protective mechanism of Simvastatin in ischemic stroke. Our data indicated that Simvastatin could protect the cerebrum from ischemic injury by upregulating the levels of PP2A and NR3A. In 2001 and 2004, Sucher NJ’s group reported that PP2A could form a stable complex with the NR3A carboxyl domain [20], [21]. Though the complex between them was not detected in the present study, a positive effect of PP2A involved in the regulatory mechanism of NR3A expression was presented.

**Figure 2:**
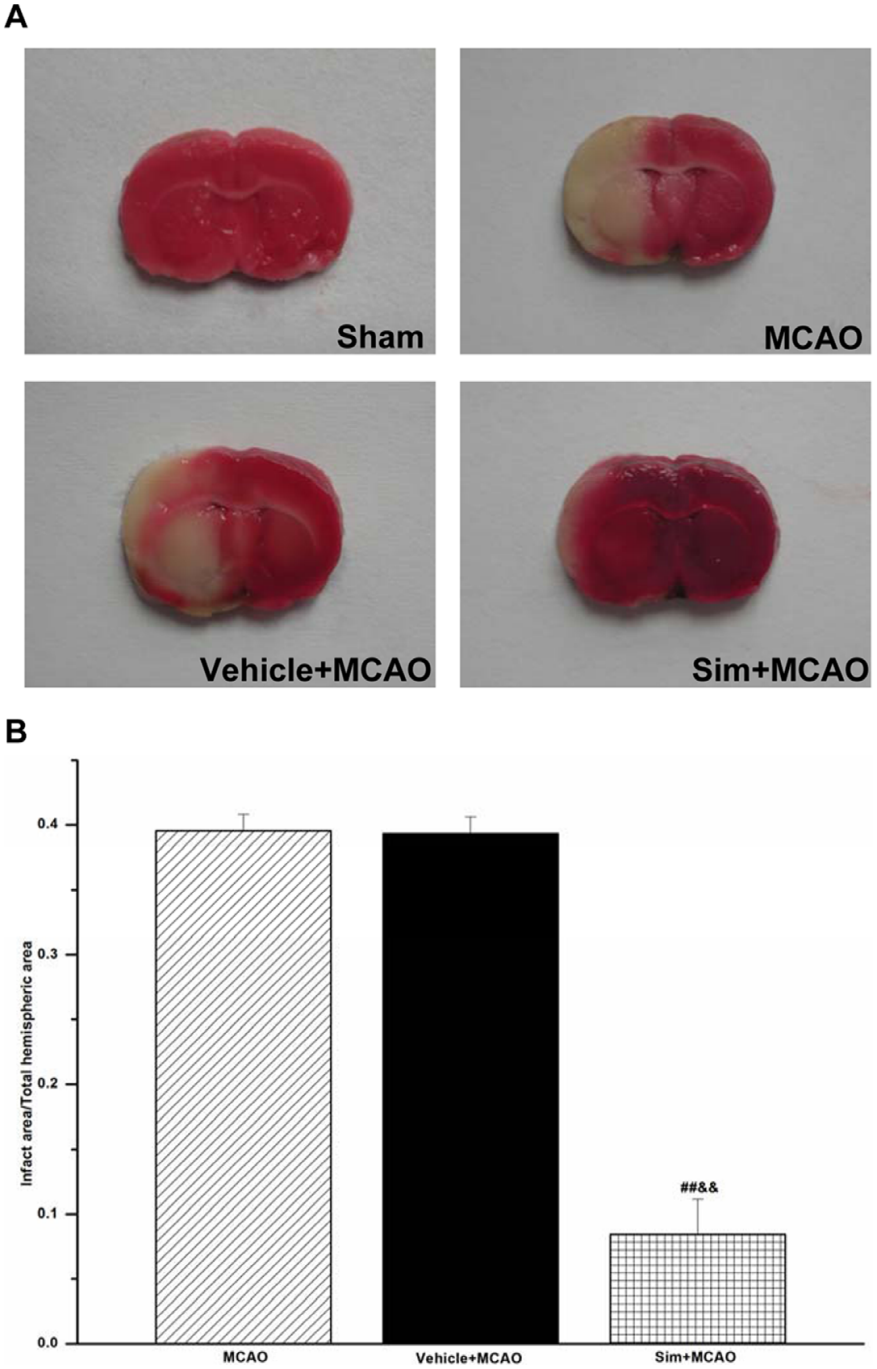
Simvastatin decreased infact area of MCAO rats. A: representative photographs of brain slices; no infarct area detected in sham group. **B:** the fourth brain slice of each rat picked out for statistical analysis; MCAO and vehicle+MCAO group showing a significant lesion, but sim+MCAO group indicating improvement in infarct area; values expressed as means ± SEM of 6 rats; ##*P*<0.01, sim+MCAO *vs*. MCAO group; &&*P*<0.01, sim+MCAO *vs*. vehicle+MCAO group. Sim, Simvastatin.

## Materials and Methods

### Ethics Statement

The current experiments conformed to the Regulations for the Administration of Affairs Concerning Experimental Animals, National Committee of Science and Technology of China and Instructive Notions with Respect to Caring for Laboratory Animals, the Ministry of Science and Technology of China, and were approved by the Ethics Committee for Experimental Research, Shanghai Medical College, Fudan University.

### Animals and Treatments

Adult male Sprague-Dawley rats weighing 210–220 g were obtained from Shanghai Laboratory Animal Center to be maintained at controlled temperature of 25°C with 12/12 light/dark cycle, during which they were allowed to have free access to food and water. The animals were randomly divided into four groups: sham, middle cerebral artery occlusion (MCAO), vehicle+MCAO, and Simvastatin (sim)+MCAO group. Simvastatin (Merck, NJ, USA) was dissolved in normal saline as vehicle, and a single dose of Simvastatin 20 mg/kg or vehicle was daily gavaged for 7 days until transient right hemisphere MCAO, which was ensured for 1 h by inserting a 4-0 surgical nylon of 18.5–19.5 mm with its tip rounded by heat from the external carotid artery into internal carotid artery until it reached the origin of the middle cerebral artery [22]. At the end of MCAO, the suture was withdrawn. After 24-h reperfusion, the animals were decapitated to collect their brains.

**Figure 3:**
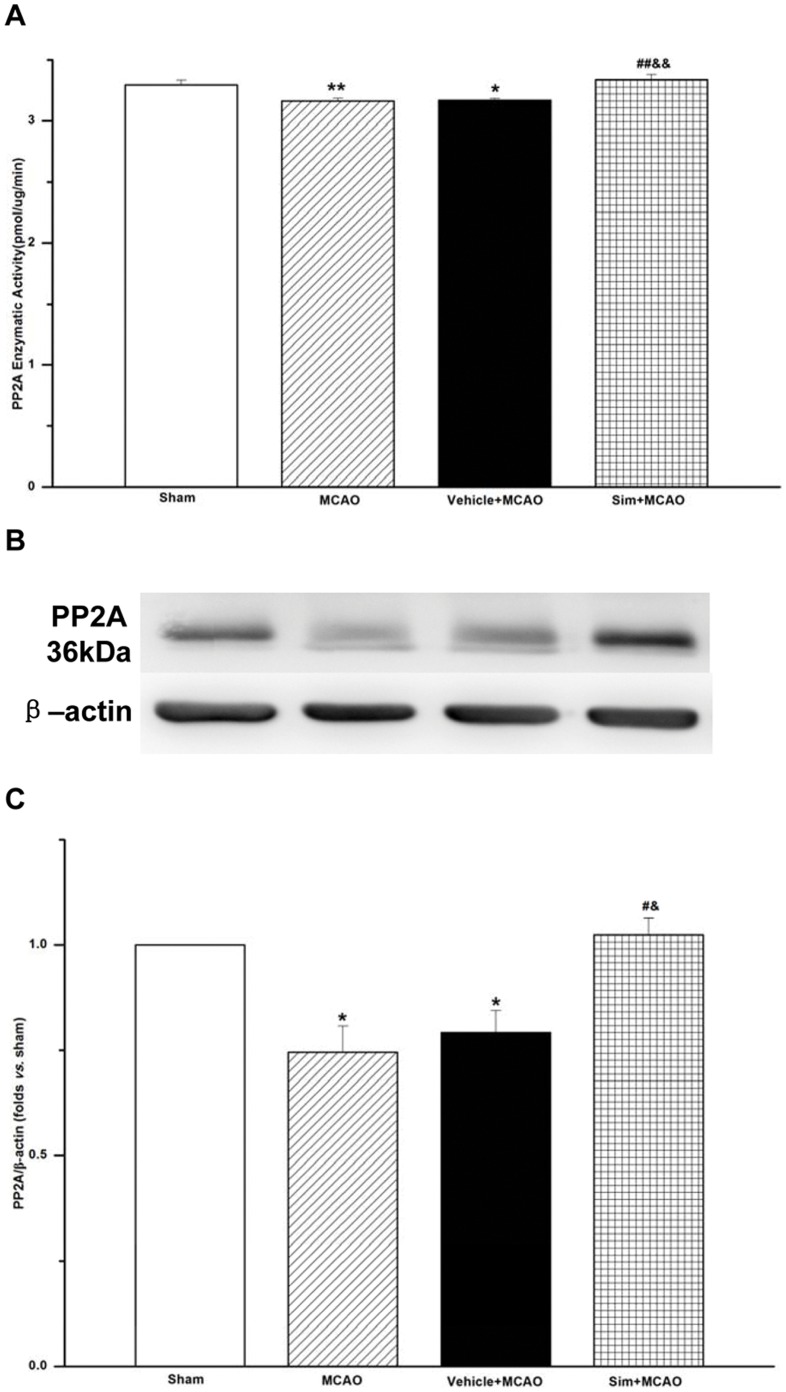
Simvastatin increased enzymatic activities and protein levels of PP2A of MCAO rats in hippocampus CA1 region. A: the enzymatic activities detected with serine/threonine phosphatase assay system; the free phosphate (pmol) calculated from the optical density of the sample at 600 nm; values expressed as means ± SEM of 6 rats; **P<0.01, MCAO *vs*. sham group; *P<0.05, vehicle+MCAO *vs*. sham group; ##*P*<0.01, sim+MCAO *vs*. MCAO group; &&*P*<0.01, sim+MCAO *vs*. vehicle+MCAO group. **B:** representative blots showing the levels of PP2A in each group. **C:** semiquantitative analysis of the levels of PP2A; values expressed as means ± SEM of 6 rats; *P<0.05, MCAO *vs*. sham group, vehicle+MCAO *vs*. sham group; #*P*<0.05, sim+MCAO *vs*. MCAO group; & *P*<0.05, sim+MCAO *vs*. vehicle+MCAO group. Sim, Simvastatin.

**Figure 4:**
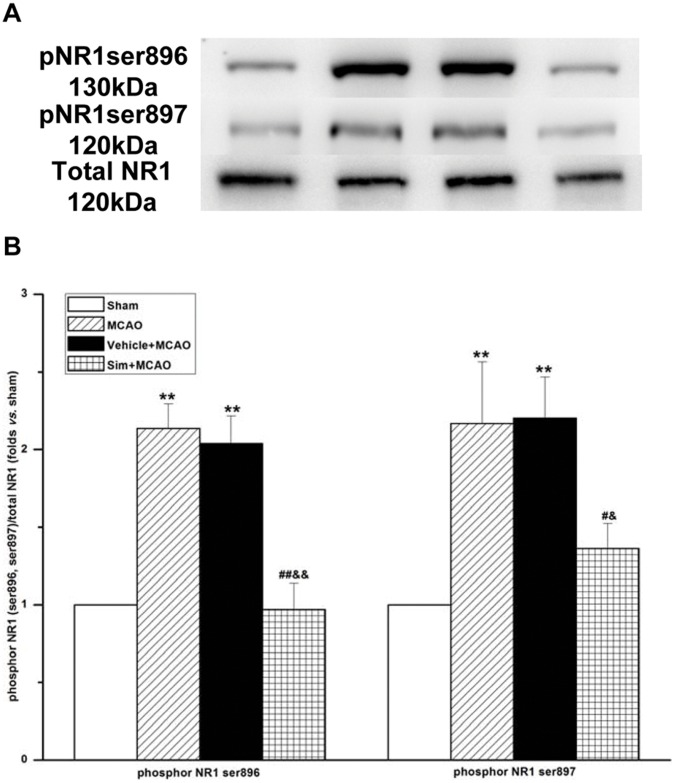
Simvastatin decreased phosphorylation of NR1 (ser896, ser897) of MCAO rats in hippocampus CA1 region. A: representative blots showing the levels of phosphrylation of NR1 (ser896, ser897) and total NR1 in each group. **B:** semiquantitative analysis of the levels of phosphrylation of NR1 (ser896, ser897) and total NR1; values expressed as means ± SEM of 5 rats; **P<0.01, MCAO *vs*. sham group, vehicle+MCAO *vs*. sham group; ##*P*<0.01, sim+MCAO *vs*. MCAO group; #*P*<0.05, sim+MCAO *vs*. MCAO group; &&*P*<0.01, sim+MCAO *vs*. vehicle+MCAO group; &*P*<0.05, sim+MCAO *vs*. vehicle+MCAO group. Sim, Simvastatin; pNR1ser896, phosphor NR1 (ser896); pNR1ser897, phosphor NR1 (ser897).

**Figure 5:**
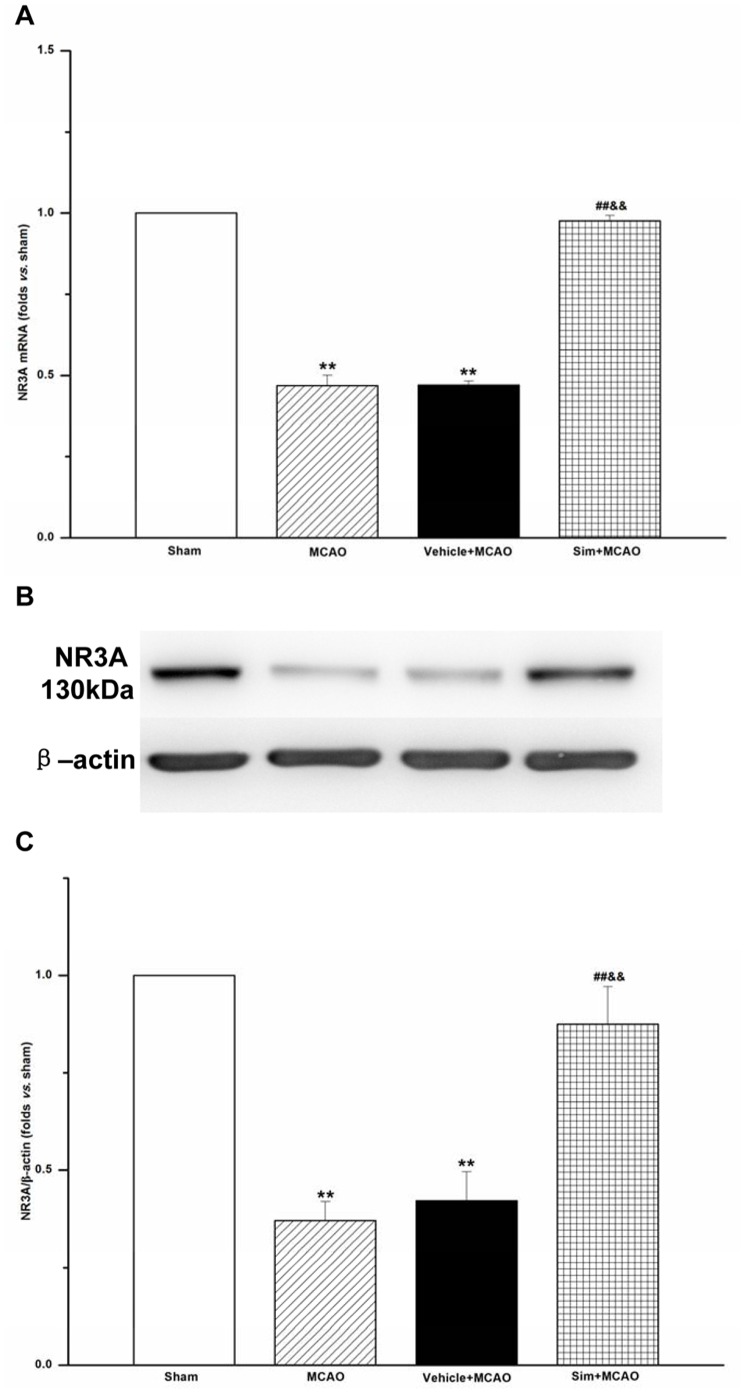
Simvastatin increased mRNA expression and protein levels of NR3A of MCAO rats in hippocampus CA1 region. A: NR3A mRNA levels analyzed by quantitative real-time PCR; values expressed as means ± SEM of 5 rats; **P<0.01, MCAO *vs*. sham group, vehicle+MCAO *vs*. sham group; ##*P*<0.01, sim+MCAO *vs*. MCAO group; &&*P*<0.01, sim+MCAO *vs*. vehicle+MCAO group. **B:** representative blots showing the levels of NR3A in each group. **C:** semiquantitative analysis of the levels of NR3A; values expressed as means ± SEM of 5 rats; **P<0.01, MCAO *vs*. sham group; vehicle+MCAO *vs*. sham group; ##*P*<0.01, sim+MCAO *vs*. MCAO group; &&*P*<0.01, sim+MCAO *vs*. vehicle+MCAO group. Sim, Simvastatin.

**Figure 6:**
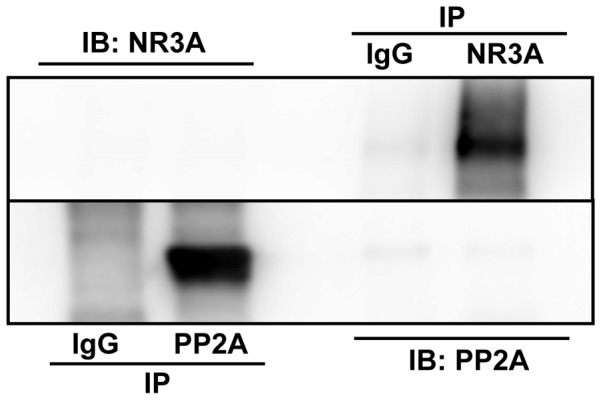
No immunocomplex observed between PP2A and NR3A of normal rats in hippocampus CA1 region. No band observed in immunoprecipitating them with anti-PP2A or anti-NR3A antibody. IP, immunoprecipitation; IB, immunoblotting.

### Microinjection

The animals were divided into two groups: sim+OA+MCAO group and sim+DMSO+MCAO group. They were gavaged with Simvastatin for 7 days, and 2 hours after the last intragastric administration, the microinjection was conducted. A burr hole, 3.5 mm posterior, 2.5 mm lateral to the bregma and 3.0 mm deep, was drilled in the skull for administration of OA (0.5 µl) dissolved in DMSO at 1 µg/µl (Sigma-Aldrich, St. Louis, MO, USA) and of DMSO in the controls (Sigma-Aldrich, St. Louis, MO, USA). The injector was retained in place for an additional 10 minutes to reduce any possible backflow of the chemical along with the injection void, and one hour later, occlusion was performed.

**Figure 7:**
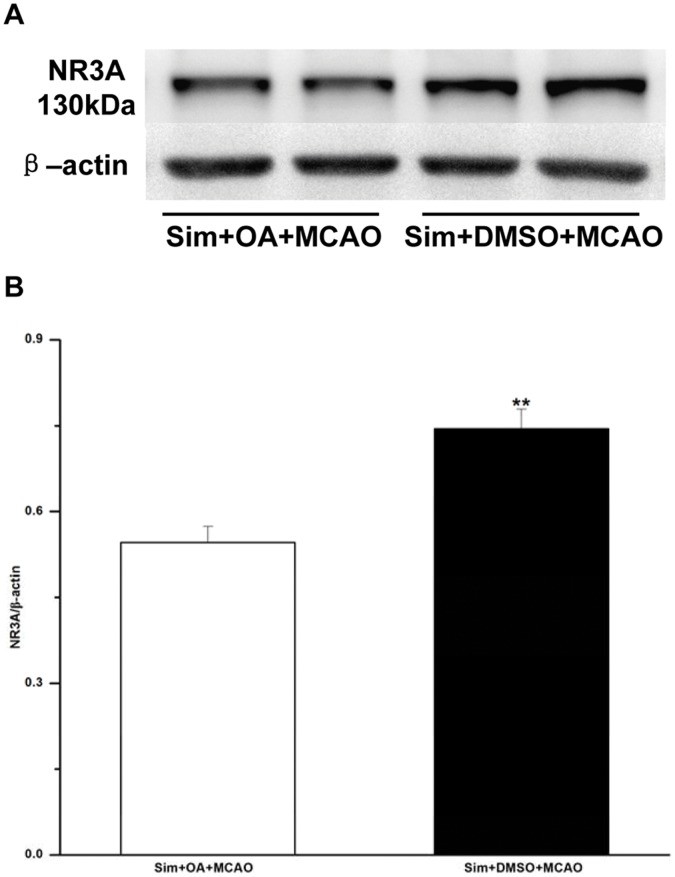
Microinjection of OA in hippocampus CA1 region of MCAO rats with Simvastatin pretreatment decreased protein levels of NR3A. **A:** representative blots showing the levels of NR3A in sim+OA+MCAO and sim+DMSO+MCAO group. **B:** semiquantitative analysis of the levels of NR3A; paired Student’s *t*-test adopted; values expressed as means ± SEM of 6 rats; **P<0.01, sim+DMSO+MCAO *vs*. sim+OA+MCAO group. OA, okadaic acid; sim, Simvastatin.

### Evaluation of Neurological Deficit and Infarct Area

The animals were evaluated for neurological deficit 24 hours after reperfusion in a double-blinded manner. Scored 0 were those which showed no observable neurological deficit; 1, which failed to extend left forepaw fully; 2, which circled to the left side; 3, which fell to the left; and 4, which did not walk spontaneously and showed a depressed level of consciousness [22]. After scoring, all test subjects were deeply anesthetized with 6% chloral hydrate and sacrificed by decapitation so that the derived brains were sectioned into 6 coronal slices in 2 mm thickness using a rodent brain matrix (RWD Life Science, Shenzhen, China), and then stained with 2% 2, 3, 5-triphenyltetrazolium chloride (TTC) (Sigma-Aldrich, St. Louis, MO, USA) at 37°C for 30 minutes in dark to detect infarct area and subsequently fixed in 4% paraformaldehyde overnight.

The stained viable brain tissues dark red and infracted those unstained, each slice was photographed under a digital camera and analyzed with Image Pro-plus 6.0 (Media Cybernetics, Sliver Spring, MD, USA). The area of infarction was calculated and expressed as a percentage of infarct area to total hemispheric one for each slice, and the fourth brain slice of each rat was adopted for statistical analysis.

### Western Blot Analysis

The tissue samples of hippocampus CA1 were homogenized in RIPA lysis buffer (Beyotime Institute of Biotechnology, Haimen, China) with protease and phosphatase inhibitor, the total protein concentration measured by bicinchoninic acid (BCA). Total protein (50 µg) was loaded into each lane onto 7.5% or 10% SDS-polyacrylamide gels. Electrophoresis was performed before the proteins were transferred to the poly-vinylidene fluoride (PVDF) membranes (Millipore, MA, USA), which were blocked with 5% non-fat milk, washed in Tris-buffered saline containing 0.1% Tween-20 (TBST), and incubated with primary antibody overnight at 4°C. The primary antibodies were as follows: mouse anti-PP2A, C subunit monoclonal antibody (1∶2000; Millipore, MA, USA), rabbit anti-NR1 monoclonal antibody (1∶1000; Cell Signaling Technology, MA, USA), rabbit anti-phospho NR1 (Ser896) polyclonal antibody (1∶1000; Millipore, MA, USA), rabbit anti-phospho NR1 (Ser897) polyclonal antibody (1∶1000; Millipore, MA, USA), rabbit anti-NR3A polyclonal antibody (1∶1000; Millipore, MA, USA), and mouse anti-β-actin polyclonal antibody (1∶3000; Beyotime Institute of Biotechnology, Haimen, China). Peroxidase-conjugated anti-rabbit (1∶1000; Millipore, MA, USA) and anti-mouse (1∶3000; Millipore, MA, USA) antibodies were used as the secondary antibody. The membranes were probed using an ECL-Plus detection kit (Beyotime Institute of Biotechnology, Haimen, China) and then scanned with Imagequant LAS4000 mini (GE Healthcare Life Sciences, CT, USA). The intensity analysis was performed using gel-pro Analyser (FURI Science & Technology, Shanghai, China).

### Co-immunoprecipitation

The tissue samples of hippocampus CA1 were homogenized in NP-40 lysis buffer (Beyotime Institute of Biotechnology, Haimen, China) with protease and phosphatase inhibitor, the total protein concentration measured by BCA. According to the protocol of immunoprecipitation kit-dynabeads protein G (Invitrogen, Oslo, Norway), 50 µl of protein G dynabeads were incubated with 1–10 µg of antibody with rotation at room temperature for 10 minutes. They were washed thrice, followed by an addition of 100 µl of tissue sample for another 10-min rotation. The complex was washed thrice and eluted with elution buffer. Goat anti-NR3A polyclonal antibody (Santa Cruz Biotechnology, CA, USA), and mouse anti-PP2A, C subunit monoclonal antibody (Millipore, MA, USA) were adopted for immunoprecipitation.

### Real-time PCR Analysis for NR3A mRNA Expression

The tissues of hippocampus CA1 were dissected and homogenized in Trizol reagent, and reverse-transcribed into first-strand cDNA using a cDNA synthesis kit. For the NR3A gene, the forward primer was 5′-CAAGTCTCACGGGTTTATGGA-3′, and the reverse primer, 5′-GTCCAGAGAAGTGCTTGATGC-3′. For the GAPDH gene, the forward primer was 5′-CCCTTCATTGACCTCAACTACATG-3′, and the reverse primer, 5′-CTTCTCCATGGTGGTGAAGAC-3′. Three-step real-time PCR of denaturing, annealing and extension reactions were performed for 40 cycles of 15 seconds at 95°C, 30 seconds at 62°C and 30 seconds at 72°C (for NR3A and GAPDH). Each sample was run and analyzed in triplicate and the Ct values for NR3A were subtracted from the Ct values of GAPDH to yield ΔCt values. The average ΔCt was calculated for the control group and this value was subtracted from the ΔCt of all other samples (including the control group). This resulted in a ΔΔCt value for all samples, which was then used to calculate the fold induction of the mRNA levels of NR3A using the formula 2^−ΔΔCt^.

### PP2A Activity Assay

PP2A activities were determined with a serine/threonine phosphatase assay system (Promega, WI, USA). The samples were homogenized in lysis buffer containing 50 mM Tris-HCl, pH 7.0, 1.0 mM PMSF, 1.0 mM EDTA, 10 mM β-mercaptoethanol, and protease inhibitors cocktail. Homogenates were centrifuged at 16,000×g for 10 minutes at 4°C, and the supernatant was prepared for PP2A activity assays and the protein concentration of supernatant were adjusted as 5 µg/35 µl before the steps as follows: the supernatant was run through Sephadex G-25 columns to remove free phosphate, 35 µl of which was then applied to a reaction premix containing phosphopeptide substrate (5 µl) and PP2A 5×reaction buffer (250 mM imidazole, pH 7.2, 1 mM EGTA, 0.1% β-mercaptoethanol, and 0.5 mg/ml BSA; 10 µl) in 96-well plates. To avoid experimental errors, all experiments were performed in triplicate. The reactions were terminated by adding 50 µl molybdate dye/additive mixture after a 15 minutes incubation at 30°C. The phosphate released from the substrate was detected by measuring the absorbance of a molybdate-malachite green-phosphate complex at 600 nm. PP2A activities were calculated by the release of phosphate per µg of protein and per minute (pmol/µg/min).

### Statistical Analysis

The data were expressed as the mean ± SEM. Statistical analyses were performed using one-way ANOVA or paired Student’s *t*-test (for sim+OA+MCAO group and sim+DMSO+MCAO group comparisons). *P* values less than 0.05 were considered significant in all cases.

## Results

### Simvastatin Decreased Neurological Deficit Score of MCAO Rats

MCAO group presented a poor neurological function when compared with sham group, which, however, showed no neurological deficit. Simvastatin pretreatment decreased the behavior scores significantly in sim+MCAO (1.38±0.18) when compared with MCAO group (2.63±0.26, *P*<0.01) (Table 1; Fig. 1).

### Simvastatin Decreased Infact Area of MCAO Rats

In sham group, no ischemic lesion area was detected, while in MCAO group, the infarct area was found to be 39.54±1.28%, which, however, significantly decreased in sim+MCAO than in MCAO group (8.45%±2.68%, *P*<0.01) (Fig. 2).

### Simvastatin Increased PP2A Levels of MCAO Rats in Hippocampus CA1 Region

Using serine/threonine phosphatase assay system and Western blot to detect the protein phosphatase activities and protein levels of PP2A in hippocampus CA1, the enzymatic activities in MCAO group (3.16±0.02) decreased when compared with that in sham group (3.30±0.04, *P*<0.01), but the administration of Simvastation increased the activities of PP2A in sim+MCAO group (3.34±0.04). Similarly, the protein level of PP2A in MCAO group was 0.75±0.06, while that increased up to 1.02±0.04 in sim+MCAO group (Fig. 3).

### Simvastatin Decreased Phosphorylation of NR1 (ser896, ser897) of MCAO Rats in Hippocampus CA1 Region

From the results, it was found that the phosphorylation of NR1 (ser896, ser897) increased markedly in MCAO group (2.13±0.16 and 2.17±0.40) in comparison with sham group, but decreased in sim+MCAO group (0.97±0.17, 1.36±0.16) (Fig. 4).

### Simvastatin Increased NR3A Levels of MCAO Rats in Hippocampus CA1 Region

With real-time PCR and Western blot analysis, it was found that mRNA expression and protein levels of NR3A in MCAO group were 0.47±0.03 and 0.37±0.05, which decreased significantly when compared with those of sham group (*P*<0.01), but the two indexes were 0.98±0.02 and 0.87±0.10 in sim+MCAO group, which increased when compared with those of MCAO group (*P*<0.01) (Fig. 5).

### No Immunocomplex Observed between PP2A and NR3A of Normal Rats in Hippocampus CA1 Region

Co-immunoprecipitation was used to observe if there were any formed complexes between PP2A and NR3A in the normal rats. The results showed no complex between them (Fig. 6).

### OA Decreased NR3A Levels of MCAO Rats with Simvastatin Pretreatment in Hippocampus CA1 Region

OA was microinjected into hippocampus CA1 region of the animals with Simvastatin pretreatment, which then received MCAO. The results showed that the protein level of NR3A in sim+OA+MCAO group (0.55±0.03) was lowered when compared with that of sim+DMSO+MCAO group (0.75±0.03, *P*<0.01) (Fig. 7).

## Discussion

Though great advances in the treatment of cardiovascular disease, the therapeutic options for acute ischemic stroke remaining limited. Tissue plasminogen activator (t-PA), the only one that was approved by the US Food and Drug Administration for use in acute ischemic stroke, was efficacious in reducing disability and saving costs for individuals. However, only a small percentage of stroke patients could be treated by t-PA. Therefore, it is urgent to search new treatments with wider therapeutic windows, less side effect, and easier and quicker administration off the hospital. Among the candidates, Statins emerged for its great potential in ischemic stroke [23]. Lidia GB et al. made use of a mata-anaylsis to evaluate the efficacy of Statins in animal stroke models, which covered Simvastatin, Atorvastatin, Rosuvastatin, etc., from which it was concluded that Simvastatin had the greatest effect on infarct volume reduction (38.18%) and neurological improvement (22.94%), and they also observed a bigger infarct reduction when a comparison was made between pre-treatment (33.5%) and post-treatment (16.02%) [24]. Similarly, our results revealed that Simvastatin significantly improved neurological deficit and reduced the infarct areas, the infarct areas in MCAO and sim+MCAO group being 39.54% and 8.45%.

Three serine sites of the NR1 subunit could be phosphorylated, and protein kinase C (PKC) phosphorylated serine residues 890 and 896 and PKA phosphorylated serine residue 897 [25]. Phosphorylation of NR1 could increase Ca^2+^ influxes and NMDAR-mediated currents [26], [27]. Previous reports have indicated that cerebral ischemia could decrease protein levels of PP2A and increase phosphorylation of the NR1, including phosphorylation sites ser890, ser896 and ser897 [28], [29], [30]. In the present study, we acquired the same results. In MCAO group, the protein expression and enzymatic activities of PP2A decreased, and the phosphorylation of NR1 (ser896, ser897) increased when compared with those of sham group. But in sim+MCAO group, the PP2A levels increased, and the phosphorylation of NR1 decreased. Yet there have been little previous experimental data suggesting a direct interaction between Simvastatin and PP2A. Our results suggested that Simvastatin administration offered partial protection for ischemic stroke by increasing the enzymatic activities of PP2A, i.e., by aiming at ser896 and ser897 to make dephosphorylation function.

In 1995, NR3A was identified by two scientific research groups, which they designated χ-1(NMDAR-L). The spatial distribution of NR3A subunit expression remains the same from postnatal day 1 to adulthood, and involves the spinal cord, brainstem, thalamus, hypothalamus, amygdala, hippocampus CA1, and cortex. During the late embryonic development, NR3A transcript levels increase and remain elevated until the second postnatal week, when levels sharply decline. The temporal expression of this subunit indicates that it may play an important role in early neuronal differentiation, migration, and synapse formation [12], [13].

Nobuki N et al. used *in vivo* models of hypoxic-ischemic to examine the effect of NR3A on cell death in retinal cultures, and they employed NR3A knockout and transgenic overexpressing mice to provide convincing evidence that ischemic-induced neuronal damage was extensive in the absence of NR3A, and cell loss was reduced in the presence of NR3A [31]. In the present study, the variation of NR3A in MCAO group suggested that brain ischemic injury induced down-regulation of NR3A in hippocampus CA1, but Simvastatin pretreatment reversed this tendency and increased NR3A levels significantly.

Limited literature is available on the alteration of NR3A in disease. In the dorsolateral prefrontal cortex (DLPFC) of schizophrenia or bipolar disorder patients, the NR3A transcript levels were reported to be elevated by 32% in schizophrenia, but decreased by 12% in bipolar disorder relative to comparison group [32]. Conversely, Henson MA et al. indicated that NR3A levels were similar in schizophrenic and control DLPFC, which, however, were lower in females when compared with males [33]. The reports suggested a close relation between NR3A and some neurological and psychiatrical diseases, and indicated some regulatory factors in modulating the expression of NR3A.

The coincidental variations of PP2A and NR3A among the four groups provided a hypothesis that there may be some correlations between PP2A and NR3A. It was reported that PP2A could form a stable complex with the NR3A carboxyl domain, which would increase the phosphatase activities of PP2A and the dephosphorylation of serine 897 of the NR1 [20], [21]. To repeat the investigation on the interaction between PP2A and NR3A, the present study adopted co-immunoprecipitation to analyze their relation. In hippocampus CA1 of the normal rat, however, no formed complex was observed between NR3A and the catalytic unit of PP2A. This disagreement could be addressed as follows: the synaptic plasma membrane and postsynaptic density fractions were employed in their study, on which NMDARs were relatively concentrated, whereas our study used the total protein of hippocampus CA1; the antibodies applied were different in the immunoprecipitation, in their study monoclonal antibody 6F9 efficient enough to recognize the core enzyme and/or holoenzyme of PP2A, and in our study monoclonal antibody-PP2A, C subunit, clone 1D6 employed, only recognizing PP2A catalytic subunit; and the formation of complex between PP2A and NR3A so transient and weak that our techniques could not detect the complex between them. To understand the exact interaction between PP2A and NR3A *in vivo*, further investigations need to be designed.

OA is known to inhibit serine/threonine protein phosphatases to different extents, PP2A the most sensitive, followed by PP1 and PP2B, and PP2C not inhibited at all. Therefore, OA may serve as a unique tool for analyzing phosphorylation-regulated systems [34]. In the present study, OA was applied to inhibiting the enzymatic activities of PP2A so as to further detect the variations of NR3A. The protein levels of NR3A in sim+OA+MCAO group were decreased when compared with that of sim+DMSO+MCAO group, which suggested that the enzymatic activities of PP2A exerted a regulative and positive effect on the expression of NR3A.

In summary, MCAO rats on Simvastatin pretreatment could markedly improve the neurological deficit, reduce brain infarct area, enhance the expression levels of PP2A and NR3A, and decrease the protein levels of phosphorylation of NR1 (ser896, ser897). Furthermore, PP2A might involve in the regulatory mechanism of NR3A expression. Our findings may potentially get a deep insight into the pleiotropic role of Simvastatin, provide a new understanding on the functional modulation of NR3A, and build a feasible basis for the protective effect of Simvastatin on brain ischemia.
